# Strength without Size Effect and Formula of Strength for Concrete and Natural Marble

**DOI:** 10.3390/ma12172685

**Published:** 2019-08-22

**Authors:** Guangchun Zhou, Jun Shi, Maohong Yu, Yu Zhang, Xiaochun Li, Yan Zhao

**Affiliations:** 1 Key Lab of Structures Dynamic Behavior and Control of China Ministry of Education, School of Civil Engineering, Harbin Institute of Technology, Harbin 150090, China; 2 Key Lab of Smart Prevention and Mitigation of Civil Engineering Disasters of the Ministry of Industry and Information Technology, Harbin Institute of Technology, Harbin 150090, China; 3 School of Transportation Science and Engineering, Harbin Institute of Technology, Harbin 150090, China; 4 Department of Civil Engineering, Xi’an Jiaotong University, Xi’an 710049, China; 5 Institute of Rock and Soil Mechanics, Chinese Academy of Sciences, Harbin 150090, China; 6 Key Laboratory of Earthquake Engineering and Engineering Vibration, Institute of Engineering Mechanics, China Earthquake Administration, Harbin 150080, China

**Keywords:** essential strength, formula of strength, stressing state, specimen, concrete, marble

## Abstract

Throughout the several-hundred-year-long history of the concept of strength, inaccurate material strength as a result of the size effect and the inconsistency of strength theories have been two continuous and challenging issues, and have even been taken to be inherent attributes of material strength. Applying the structural stressing state theory and method, this study experimentally investigates the uniaxial load-bearing process of concrete specimens and reveals their stressing state mutation features at specific load levels. Exploration of this general feature resulted in the discovery of essential strength, which is basically without size effect. Then, biaxial and triaxial experiments with concrete specimens were conducted in order to obtain the results for various combinations of principal stresses on essential strength. Consequently, according to Yu’s unified strength theory, the formula for strength of concrete was determined by fitting the relation between the combined principal stresses and the essential strength, which was verified by experiments carried out using natural marble specimens. Essential strength could promote the accuracy of strength indices, and the formula for strength might replace the existing strength theories for brittle materials. The initial solution of these two classic issues could make a new contribution to Yu’s unified strength theory and its final goal, promoting related research on material strength and leading to a more rational use of material strength in practical engineering.

## 1. Introduction

Since the concept of strength was proposed by da Vinci in the 1500s and Galileo in 1638, scientists from around the world have developed up to one hundred theories addressing issues related to strength. Yu reviewed 1163 references and summarized the research achievements related to strength theories/criteria since 1638 [1], particularly over the last 100 years [2], and suggested that two issues had accompanied the concept of strength throughout its history:

The first issue was the inaccuracy of material strength determinations resulting from the specimen’s size effect (abbr. size effect). Early in the 16th century, da Vinci realized the effect of size on material strength. Subsequently, researchers were forced to acknowledge that the size effect was unavoidable, such that it seemed impossible to obtain the accurate strength for a material. This impossibility was even assumed to be an inherent property of material strength. Accordingly, many countries have developed individual regulations for obtaining material strength indices and making them available for related research and engineering applications [3,4,5]. Thus, it was commonly thought that research into uniaxial material strength indices had come to an end, and most researchers turned to other issues related to material strength, such as impact [4], cyclic [6] and tensile/compressive [3,6,7,8] performances, and constitutive/failure models [1,9,10] (size effect involved [3]) for various concrete specimens [1,3,4,6,7,9]. 

The second issue was that up to one hundred of these strength theories and criteria were basically inconsistent in terms of forms of expression or physical implications [11,12], and material strength is certainly related to the complex stress states of unit bodies in reality. Now, the classic and most commonly applied strength theories or criteria are the 1st–4th strength theories. The 1st and 2nd strength theories were proposed before 1900, based on Rankine’s strength criterion [11] and Mariotte and Saint-Venant’s strength criteria [12,13], respectively. During the last century, the 3rd strength theory was derived from Tresca and von Mises’ strength criteria [14,15], and the 4th strength theory was derived from von Mises’ strength criterion [15]. One recent achievement was the unified strength theory proposed based on Yu’s strength criterion [16], which is the closest to the essence of material strength, as its expression is able to cover the greatest number of strength theories and criteria. However, early in the 20th century, a number of famous scientists, including Voigt and Mohr, believed that it would be impossible to develop a single strength theory that would cover various materials [6,15], in other words, a single strength criterion did not exist for various materials. Until now, it has commonly been taken for granted that there is no definite relation between material strength and combined principal stresses. 

As a result, these two issues have even been regarded as intrinsic attributes of material strength [9,17,18,19,20,21,22,23,24,25,26,27]. Furthermore, these innate attributes were essentially a result of a common cause: size effect, i.e., all research on material strength was inevitably carried out taking account of the size effect. In addition, variations in the manufacture of material and the testing of material strength were commonly thought to be the reason for inaccuracies in material strength. However, structural stressing state theory and its applications have led us to investigate these innate attributes as follows:

Essentially, a material strength depends on the maximum uniaxial stress magnitude that the unit body can withstand [28]. In the existing design codes of concrete, σ was derived based on the ultimate loads of specimens and included the size effect. However, it is doubtful that this can explain the effect of the specimen’s size on the material strength, because the strength of any unit body in a specimen will fail as long as its uniaxial stress reaches the definite value σ, no matter the loading case or the size of the specimen. The material strength defined by the unit body should be definite and independent of the sizes and shapes of specimens, like the specific gravity of a substance. It could be seen from experimental observations of concrete specimens that specimens were severely broken at their ultimate load, and that most unit bodies were in complex (non-uniaxial) and non-identical stress states. This fact implies that ultimate loads and yield loads of specimens might be inappropriate for determining uniaxial material strength [3,4,5]. The accurate material strength should exist in the uniaxial load-bearing process of the specimen somewhere and there the unit bodies in the specimen will lose their identical and uniaxial stress states or their ability to withstand average uniaxial stress. Hence, this paper experimentally investigates the uniaxial stress process of concrete specimens with different sizes based on the structural stressing state theory and method, leading to the discovery of essential strength, basically without a size effect. 

Strength theories/criteria have generally been derived based on mechanical models and mathematical equations with some assumptions, including the material properties and forms of failure, rather than from totally experimental results. Even for the classic single-shear criterion, twin-shear criterion and unified yield criteria, a linear and/or incomplete basis has been taken in order to express material strength with naturally nonlinear properties. Also, although the unified strength theory could unify strength theories/criteria within a single expression, it expresses the individual strength criteria through adjustable parameters. The discovery of essential strength implies that there should be a definite formula describing the relationship between essential strength and combined principal stresses. Hence, this study investigates the experimental data of the biaxial and triaxial stressing processes of concrete specimens using the proposed methods, and obtains the results of combinations of principal stresses with respect to the essential strength of concrete. Consequently, the formula for the strength of concrete was determined by fitting the relation between essential strength and the combination of principal stresses. The formula for strength was verified through biaxial and triaxial experiments using natural marble specimens. 

## 2. Structural Stressing State Concept and Method

### 2.1. Concept and Modeling of a Specimen’s Stressing State

The natural law stating that quantitative change leads to qualitative change in a system suggests that the working behavior of a physical system when encountering an input action will definitely present a qualitative change in response to the quantitative change [29]. This led to the development of the structural stressing state theory, which is able to reveal the essential working state features of structures subjected to a full loading process [30]. This study applies the structural stressing state concept and method in order to model the load-bearing process of specimens for concrete strength. In addition, the stressing states of specimens are expressed as the generalized strain energy density (GSED) distribution mode/pattern of the unit bodies in the specimen [31]. The stressing state mode can reflect two basic characteristics: (i) the stressing state of the specimen is stable and fixed for a loading case and within a load magnitude; (ii) the change of the stressing state only corresponds to a specific load, which is defined as the failure load of the specimen. It needs to be emphasized again that these two characteristics are embodiments of the natural law that quantitative changes in a system lead to qualitative changes, rather than accidental or random phenomena. Ordinarily, the stressing state mode of a specimen is expressed as the vector or matrix $S_{j}$ consisting of GSED values (*e_ij_*) of all or some typical measured points to the *j*th load value $F_{j}$

(1) $$S_{j}={\left[e_{1j},e_{2j},\ldots ,e_{nj}\right]}^{T},\;e_{ij}=\int_{0}^{\varepsilon_{ij}}\sigma_{ij}d\varepsilon ,\;E_{j}=\sum e_{ij}$$

where $\varepsilon_{ij}$ and $\sigma_{ij}$ are the *i*th strain and stress values for the *j*th load $F_{j}$; *n* is the number of unit bodies (or measured points). Correspondingly, the parameter characterizing $S_{j}$ is defined as $E_{j}$, which is the GSED sum of the elements in $S_{j}$. The evolution of $E_{j}$ with load increase will reflect the stressing state change of the specimen. Actually, the *E_j_*-*F_j_* (abbr. E−F) curve will embody the qualitative mutation of $S_{j}$ when the quantitative change in **S***_j_* reaches a certain extent, which can be detected by the Mann-Kendall (M-K) criterion below. 

### 2.2. The M-K Criterion

The stressing state mutation feature of the specimen is surely embodied in the curve, which is an objective law of structural working behavior. However, the empirical or intuitive judgment of mutation features could be somewhat subjective, or even misleading; therefore, the statistical M-K criterion is used to detect mutations in the E−F curve. There are two reasons contributing to the choice of the M-K criterion [32,33,34,35]: (i) its function of detecting trend changes is only applicable to identifying the location at which the specimen’s stressing state mutates; (ii) it is unnecessary for numerical samples to obey a definite distribution or to be concerned about some abnormal interferences, making it suitable for experimental data that do not strictly conform to a definite distribution and contain some abnormal values. Additionally, although the M-K criterion requires the independence of the numerical sequence, the experimental data of specimens can also be investigated using the M-K criterion. This is because the relevant and independent ingredients coexist in the response of the specimen, and units far away from each other have little spatial relevance or mutual effects, resulting in the independence of the ingredients in the experimental data. Also, the innate randomness in the experimental model and material properties leads to some independent ingredients as well. Hence, the stressing state mutation of the specimen can be detected on the basis of the M-K criterion. The procedure of the M-K criterion is: 

For the numerical sequence {*E*(*i*)} (the load step *i* = 1, 2,…, *n*), a statistical quantity *d_k_* at the *k*th load step can be defined as

(2) $$d_{k}=\sum_{i}^{k}m_{i}(2\leq k\leq n),\;m_{i}=\left\{ \begin{array}{ll} +1 & E\prime (i)>E\prime (i)(1\leq j\leq i)\\ 0 & \mathrm{otherwise} \end{array}\right.$$

where *m_i_* is the cumulative number of the samples; “+1” adds one more to the present value if the inequality on the right side is satisfied for the *j*th comparison. Calculate the mean value and variance of the statistical quantity *d_k_*

(3) $$E(d_{k})=k(k-1)/4(2\leq k\leq n),\;Var(d_{k})=k(k-1)(2k+5)/72(2\leq k\leq n)$$

Then, a new statistical quantity *UF_k_* is defined by

(4) $$UF_{k}=\left\{ \begin{array}{cc} 0 & k=1\\ d_{k}-E(d_{k})/\sqrt{Var(d_{k})\;} & 2\leq k\leq n \end{array}\right.$$

and the *UF_k_*-*F* curve can be plotted.

For the inverse sequence of {*E*(*j*)} (the load step *j* = *n*, *n* − 1, …, 1), the same steps from Equation (2) to Equation (4) are carried out to derive the *UB_k_*-*F* curve. 

Finally, the intersection of the *UF_k_*-*F* and *UB_k_*-*F* curves defines the characteristic point of the *E*-*F* curve, i.e., the mutation point of the structural stressing state. 

## 3. Essential Strength of Concrete 

### 3.1. Concrete Specimens and Their Uniaxial Experiment

In accordance with the structural stressing state concept and method described above, this study conducted uniaxial and compressive experiments using concrete specimens. Cube, prism and cylinder concrete specimens with different sizes were carefully made to macroscopically embody homogeneous and isotropic properties, as listed in Table 1. The concrete No. was C40 with sand content: 0.31, water-cement ratio: 0.32, cement:sand:stone (1:1.117:1.863), curing condition (20 ± 2) °C, relative humidity > 90% and 28d, with reference to the code for design of concrete structures of China [36,37,38,39,40,41,42,43,44,45,46].

**Figure 1:**
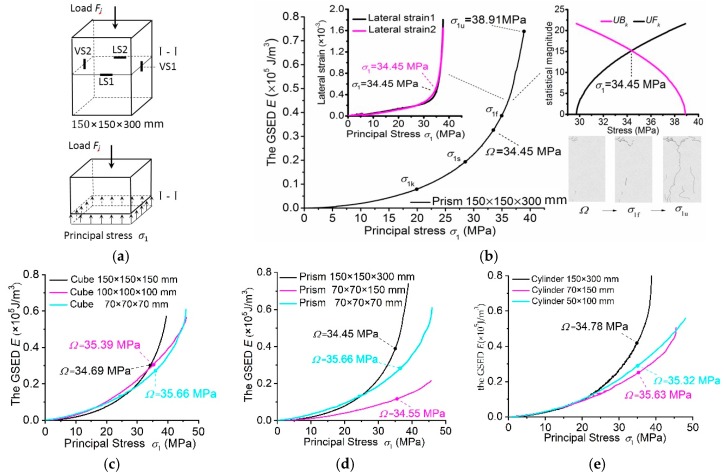
Investigation of the uniaxial stressing states of specimens. (**a**) The stress state of a prism specimen on the middle cross-section I-I and the lateral/vertical strain gauges LS/VS; (**b**) the $E-\sigma_{1}$ curve of the concrete specimen and its characteristic points $\sigma_{1k}$, $\sigma_{1s}$ and Ω detected by the M-K criterion, as well as the average ultimate stress $\sigma_{1u}$; (**c**–**e**) The characteristic points (Ω) in the $E-\sigma_{1}$ curves of cube, prism and cylinder concrete specimens at their failure loads. Compressive stress is positive here.

At least three specimens with the same size and shape were tested. The experimental apparatus was set up so that the acting displacement (load) increment would be at a given rate of 1.0 mm/min. The experiment recorded the displacement at the top side and the cracking profile on the vertical side. For the concrete specimens that were 150 mm in width or diameter, the vertical and horizontal strains were also recorded, as the strain gauges could be placed on their vertical sides, as shown in Figure 1a.

### 3.2. Investigation into the Uniaxial Stressing States of Specimens

For the prism specimen shown as an example in Figure 1a, $F_{j}$ is the resultant force of a uniformly distributed load acting on the top side of the specimen at the *j*th load. $\sigma_{1}$ is the average stress on the cross-section I-I at *F_j_*. Using Equation (1), the vertical strains (Figure 1b) measured at the *j*th load can be expressed as the GSED values, and their sum is denoted as *E* to characterize the stressing state of the specimen. Thus, the $E-\sigma_{1}$ curve can be plotted to reflect stressing state evolution. For convenience, compressive stress is set as scalar (positive sign) here. 

Then, the M-K criterion detects the mutation points $\sigma_{1k}$, $\sigma_{1s}$ and Ω in the $E-\sigma_{1}$ curve, together with observing the strain-developing tendency and the cracking profile. $\sigma_{1k}$ in segment 0~$\sigma_{1u}$ is the turning point at which the specimen shifts from a linearly elastic working state to an elastic one. $\sigma_{1s}$ in segment $\sigma_{1k}$~$\sigma_{1u}$ is the turning point at which the specimen shifts from an elastic working state to an elastic-plastic one. In particular, Ω in segment $\sigma_{1s}$~$\sigma_{1u}$ is the turning point at which the specimen shifts from the elastic-plastic working state to the failure one. According to structural stressing state theory, Ω is the starting point of the specimen’s failure process, at which the specimen enters the developing failure state, until it reaches the ultimate state $\sigma_{1u}$. It can be observed from the lateral strain $\sigma_{1}$ curves (Figure 1b) that the lateral strains maintain quite a linear increase and do not exhibit any mutations, even at $\sigma_{1k}$ and $\sigma_{1s}$ before Ω, indicating that the specimen maintains a stable uniaxial stressing state. After Ω, the lateral strains increase sharply, implying that the stable uniaxial stressing state of the specimen has failed or has changed to another state. Correspondingly, based on the cracking pictures of the specimen (Figure 1b), it can be seen that only a few tiny cracks occur at Ω; after Ω, the cracks quickly propagate with load increase, as shown in the crack profiles, to $\sigma_{1f}$ and $\sigma_{1u}$. Similarly, the $E-\sigma_{1}$ curves of the cube and prism specimens with different sizes (unit: mm) can be plotted, and the M-K criterion is able to distinguish the mutation points (Ω) in the curves, as shown in Figure 1c,d. Evidently, the mutation points (Ω) correspond to nearly the same average stress value, around Ω = 35 MPa, with small errors within 0.5 MPa. This evidence suggests that the concrete strength could be determined basically without size effect if Ω were defined as the concrete strength index.

### 3.3. Essential Strength of Concrete without Size Effect

Researchers on material strength have been pursuing accurate material strength indices for a long time. At present, the sizes of the standard specimens for deriving material strength are based on engineering practice and experimental achievements that have, to a great extent, approached the essence of material strength. Therefore, the standard specimens make is possible to reflect material strength at a certain scale, but at present, the inaccurate working state of the specimen is used for deriving the material strength, leading to a considerable size effect and variation in strength values. As per the definition, a material’s strength is the maximum uniaxial stress that can be withstood by the unit [28,47], i.e., the material strength should be the average uniaxial stress of the units in the specimen at which they lose their consistent and uniaxial stressing states, or enter complex and inconsistent stress states. However, the existing material strength is derived at the end point of the failure process of the specimen, where units are in their complex and inconsistent stress states, rather than at the starting point, where units have only lost their consistent and uniaxial stress states. Typically, the strength of concrete (σ) is determined using the ultimate load or the limited residual strain of the specimen, whereby the stress states of the unit bodies are inconsistent or non-identical. As a result, the σ for the ending points of the $E-\sigma_{1}$ curves implies a considerable size effect and degree of variation (Figure 1c,d). In this study, the failure load of the specimen can be determined by the M-K criterion. This failure load can be used to derive the concrete’s strength using the small size effect present with the sizes of standard specimens, i.e., the concrete strength defined by Ω is basically constant (Ω = 35 MPa) and basically unrelated to the sizes of the specimens (Figure 1c,d). Additionally, Ω causes the concrete strength to be closer to the essence of material strength, which should be unrelated to the size of the specimen, like with specific gravity. In other words, the stressing state mutation of the specimen at its failure load could be small relative to the variation in material structure, specimen configuration and experimental errors. Therefore, concrete strength should be defined at the failure loads of the specimens instead of at their ultimate loads, and Ω is referred to as the essential strength. While concrete is a highly typical brittle material, other brittle materials will have their own essential strengths.

In contrast with the ultimate load of the specimen being used to derive the concrete strength, the stressing state mutation of the specimens reveals the gap between the failure load and the ultimate load. Actually, the two ends of this gap are the starting point (the failure load) and the end point (the ultimate load) in the failure process of the specimen under the uniaxial loading case. The starting point reflects the maximum uniaxial stress-bearing capacity of a unit body, which can be used to derive the essential concrete strength, largely without size effect, thus meeting the definition of material strength; the ending point reflects the maximum complex stress-bearing capacity of a unit body, and can be used to derive the material strength with the size effect, which does not entirely meet the definition of material strength. 

## 4. Unified Formula of Strength for Concrete

### 4.1. Investigation into Biaxial and Triaxial Stressing States of Concrete Specimens

**Figure 2:**
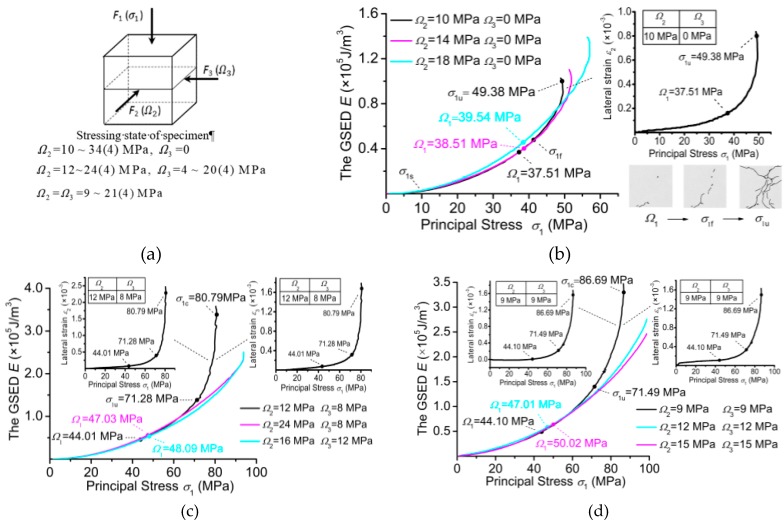
The $E-\sigma_{1}$ curves and the characteristic points. (**a**) The triaxial stress state of a specimen. (**b**) The $E-\sigma_{1}$ curves of the biaxial specimens with their characteristic points $\sigma_{1s}$ and $\Omega_{1}$, and the lateral strain $\sigma_{1}$ curve ($\Omega_{2}\;=\;10\;\mathrm{MPa},\;\Omega_{3}\;=\;0$) with the characteristic points ($\Omega_{1}$, $\sigma_{1u}$ and $\sigma_{1u}$ close to $\Omega_{1}$) to their cracking profiles. (**c**,**d**) The $E-\sigma_{1}$ curves of the triaxial specimens, and two lateral strain-$\sigma_{1}$ curves ($\Omega_{2}\;=\;9\;\mathrm{MPa},\;\Omega_{3}\;=\;8\;\mathrm{MPa}$; $\Omega_{2}\;=\;\Omega_{3}\;=\;9\;\mathrm{MPa}$) with their characteristic points ($\Omega_{1}$, $\sigma_{1c}$).

The discovery of essential strength inspired us to think that there might be a definite relation between essential strength and the combined principal stresses. Accordingly, biaxial and triaxial experiments were carried out in order to obtain the combinations of principal stresses present at the failure loads of the concrete specimens. Figure 2a shows the size of the specimens (100 mm × 100 mm × 100 mm) and the loading cases corresponding to the combined principal stresses (compressive stress is positive). The loading apparatus firstly exerted the given confining forces $F_{2}$ and $F_{3}$ on the vertical sides of the specimen, making it possible to derive the second and third principal stresses $\Omega_{2}$ and $\Omega_{3}$. Then, based on the active force *F*_1_ on the top side of the specimen, the first principal stress *σ*_1_ was derived. $\sigma_{1}$ was denoted as *Ω*_1_ at the failure load of the specimen. Figure 2a also shows the combinations of principal stresses ($\Omega_{2}=0$, $\Omega_{3}\neq 0$) and ($\Omega_{2}\neq 0$, $\Omega_{3}\neq 0$) at the given confining load $F_{2}$ or $F_{3}$, whereby the number in brackets is the interval of the serial principal stresses $\Omega_{2}$ and $\Omega_{3}$. The experimental displacements and strains along the three loading directions are applied to plot the $E-\sigma_{1}$ curves for the biaxial stress states (*Ω*_2_ ≠ 0, *Ω*_3_ = 0) in Figure 2b and for the triaxial stress states ($\Omega_{2}\neq 0$, $\Omega_{3}\neq 0$) in Figure 2c,d. The point at about $\sigma_{1s}\;=\;10\;\mathrm{MPa}$ is set as the starting point of the curve segment to apply the M-K criterion, because the load $F_{2}$ or the loads $F_{2}$ and $F_{3}$, initially confined to the specimen, could cover up the acting effect of $F_{1}$ (as $\sigma_{1}$) until $\sigma_{1s}$. For the biaxial experiment in Figure 2b, the end of the $E-\sigma_{1}$ curve is also referred to as $\sigma_{1u}$, corresponding to the ultimate load of the specimen judged from its experimental working state. Thus, from $\sigma_{1s}$ to $\sigma_{1u}$, the mutation points ($\Omega_{1}$) at the failure loads of the biaxial specimens can be detected using the M-K criterion. In addition, from the cracking pictures of the specimen, it can be observed that some micro-cracks occur at $\Omega_{1}$ (the picture to $\Omega_{1}$), but the corresponding lateral strain remains at relatively low values, with a stable increase from $\sigma_{1s}$ to $\Omega_{1}$. After $\Omega_{1}$, the cracks propagate quickly until the ultimate stressing state of the specimen (the pictures at $\sigma_{1f}$ and $\sigma_{1u}$). It should be noted that the cracks in the triaxial specimen propagate until its collapse. However, from σ1u on, the apparatus parts play a considerable constraint role in the collapsing state of the triaxial specimen, such that the specimen can still stand a little more load than $\sigma_{1u}$, while the corresponding strain increases steeply. So $\sigma_{1u}$ is still taken as the end of the $E-\sigma_{1}$ curve for the triaxial specimen. Then, from $\sigma_{1s}$ to $\sigma_{1u}$, the M-K criterion detects the failure load of the specimen together with the judgments of lateral strain and cracking profiles (Figure 2b–d).

Thus, a combination of principal stresses ($\Omega_{1}$, $\Omega_{2}$, $\Omega_{3}$) can be obtained at the failure load of the biaxial or the triaxial specimen, i.e., $\Omega_{2}$ and $\Omega_{3}$ are derived from the given confining forces $F_{2}$ and $F_{3}$, and $\Omega_{1}$ from the failure load of the specimen. Table 2 lists the typical combinations of principal stresses (compressive stress is positive). The biaxial combined principal stresses are $\Omega_{1}\neq 0$, $\Omega_{2}\neq 0$ and $\Omega_{3}=0$; the triaxial combined principal stresses are $\Omega_{1}\neq 0$, $\Omega_{2}\neq 0$ and $\Omega_{3}\neq 0$. Furthermore, Table 2 also lists the data for verifying the following formula for strength. 

### 4.2. The Formula for the Relationship between Essential Strength and Combined Principal Stresses

The material strength under a combination of principal stresses must be the same as the uniaxial material strength, i.e., the equivalent stress of the combined principal stresses must be equal to the uniaxial material strength [1,38]. Table 2 provides the data to fit/formulate the relation between the essential strength and the corresponding combined principal stresses. Here, it should be stated that according to material mechanics, the concrete could be a macroscopically homogeneous and isotropic material; therefore, the direction of the main axis for material strength needs to be consistent with the first principal stress $\Omega_{1}$ or the equivalent stress $\Omega_{r}$. With reference to the unified expression of equivalent stress in Yu’s unified strength theory [1], Equation (5) is defined for expressing the general relationship between equivalent stress and essential strength

(5) $$\Omega_{1}-a\Omega_{2}-b\Omega_{3}=\Omega$$

in which *a* and *b* are the coefficients weighing the contributions of $\Omega_{2}$ and $\Omega_{3}$ to the equivalent stress $\Omega_{r}=\Omega_{1}-a\Omega_{2}-b\Omega_{3}$ in the direction of $\Omega_{1}$; Ω is the essential strength and has the same sign as $\Omega_{1}$. $\Omega_{r}$ represents the collective effect of three principal stresses ($\Omega_{1}$, $\Omega_{2}$, $\Omega_{3}$) on the essential strength (Ω). In fitting Equation (5) using the data in Table 2, any two different combinations of principal stresses can be substituted into Equation (5) in order to form simultaneous equations, and these equations can then be solved in order to obtain *a* and *b*. The fitted *a* and *b* values range from 0.2 to 0.3 and from 0.7 to 0.8, respectively. The averages of *a* and *b* are 1/4 and 3/4, with fairly small variations of −0.0032 and 0.0047. The weight coefficients *a* and *b* are basically constant for the essential strength and the combined principal stresses. Thus, a precise and definite relationship between essential strength (Ω) and combined principal stresses ($\Omega_{1}$, $\Omega_{2}$, $\Omega_{3}$) can be defined as

(6) $$\Omega_{1}-\frac{1}{4}\Omega_{2}-\frac{3}{4}\Omega_{3}=\Omega$$

In Equation (6), both $\Omega_{1}$ ($\Omega_{r}$) and Ω can be compressive or tensional. Equation (6) adopts the following rule for sorting principal stresses: compressive cases use a positive sign for compressive principal stresses, and a negative sign for tensile principal stresses. Contrariwise, tensional cases take a positive sign for tensional principal stresses and a negative sign for compressive principal stresses. Thus, the relationship $\Omega_{1}\geq \Omega_{2}\geq \Omega_{3}$ is applicable in cases of compression and tension. 

Equation (6) is referred to as a formula for strength for two reasons: (1) the formula is directly derived by fitting experimental data, without the reliance on assumptions (elastic or plastic basis, type of material, type of failure) generally necessary for a strength theory; and (2) importantly, the essential strength and the formula for strength are derived from the consistent stressing state features (mutation) of the specimens, which is the embodiment of the natural law stating that quantitative change in a system will lead to qualitative change in that system.

## 5. Verification of the Formula of Strength

### 5.1. The Definite Relation between Uniaxial Strength and Shear Strength

There are two fundamental material strengths, uniaxial (tensional or compressive) strength σ and shear strength τ. However, the relation between σ and τ has thus far been uncertain, and this is a classic issue. Equation (6) addresses this issue on the basis of essential strength. 

For a pure shear case for a unit body, if the shear stress is *τ*, the principal stresses $\sigma_{1}=\tau$, *σ*_2_ = 0 and *σ*_3_ = −*τ* [48]. Then, substituting the combined principal stresses into the left of Equation (6) results in the equivalent stress $\sigma_{r}=7\tau /4$. When $\sigma_{r}$ reaches Ω (essential strength), $\tau_{\Omega }$ at this moment is defined as the shear strength. Thus, a definite relation can be derived as

(7) $$\tau_{\Omega }=\frac{4}{7}\Omega$$

Equation (7) evidently presents two essential characteristics of material strength: (i) the most fundamental material strength is *Ω* or *τ_Ω_*, as they can be definitely expressed by each other, implying the unification of *Ω* and *τ_Ω_* for a material; (ii) $\tau_{\Omega }$ is also essential strength according to essential strength *Ω*.

### 5.2. Rationality of Weight Coefficients in the Formula for Strength

**Figure 3:**
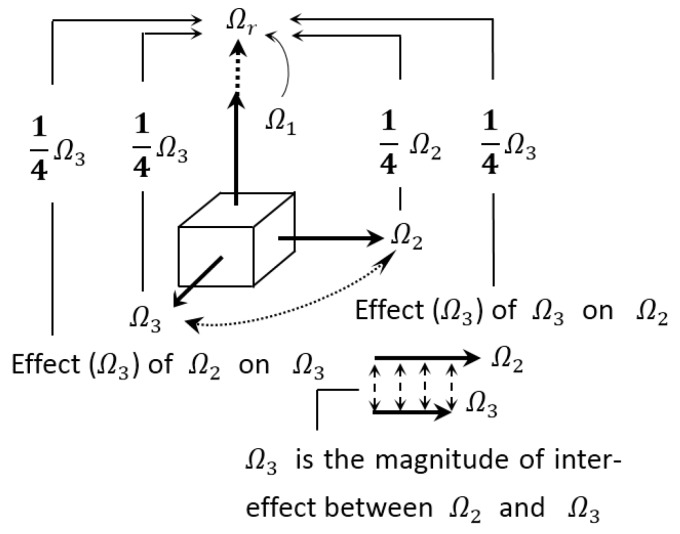
The contributions of principal stresses $\Omega_{2}$, $\Omega_{3}$ and their interactions ($\Omega_{3}$) to the equivalent stress $\Omega_{r}$ through the identical weights (1/4).

In Equation (6), the weight coefficients *a* and *b* satisfy a+b=1, which is not the preset result, but rather the one fitted by the experimental data (Table 2). The coefficients in Equation (6) show the homogeneous contributions of $\Omega_{2}$, $\Omega_{3}$ and their interactions ($\Omega_{3}$) to $\Omega_{r}$ by the identical weights (1/4), i.e., $\Omega_{r}=\Omega_{1}-[(1/4)\Omega_{2}+(1/4)\Omega_{3}+(1/4)\Omega_{3}+(1/4)\Omega_{3}]=\Omega_{1}-(1/4)\Omega_{2}-(3/4)\Omega_{3}$. Any other weight coefficients apart from a=1/4 and b=3/4 are not able to reflect the homogeneity. Figure 3 explains why $\Omega_{3}$, rather than $\Omega_{2}$, embodies the contribution of the action of $\Omega_{2}$ on $\Omega_{3}$ (or $\Omega_{3}$ on $\Omega_{2}$) to the equivalent stress $\Omega_{r}$. This is because $\Omega_{2}$ is greater than *Ω*3, and their difference is not of sufficient scale whereby $\Omega_{3}$ would be able to impose its effect on $\Omega_{2}$ or $\Omega_{2}$ could impose its effect on $\Omega_{3}$. Therefore, the interaction between $\Omega_{2}$ and $\Omega_{3}$ has the same scope, depending on that with the lower value, complying with Newton’s third law of action and reaction. 

In the case of $\Omega_{1}=\Omega_{2}=\Omega_{3}$, a physical phenomenon occurs in which strength failure cannot take place ($\Omega_{r}=0$), no matter how great the three equal principal stresses are. Equation (6) reflects this physical phenomenon by means of weight coefficients (a+b=1); otherwise, as long as (*a* + *b*) ≠ 1, Equation (6) fails with respect to this physical phenomenon. In the case of $\Omega_{2}^{+}=-\Omega_{3}^{-}$ (where “+” and “−” indicate tensional and compressive principal stresses), the weight coefficients a=1/4 and b=3/4 reflect $\Omega_{2}^{+}$ and $\Omega_{3}^{-}$ having a nonzero contribution to *Ω_r_*, which compensates for the sense that $\Omega_{2}^{+}$ and $\Omega_{3}^{-}$ should have a zero contribution to as a result of $\Omega_{2}^{+}=-\Omega_{3}^{-}$ (e.g., the 2nd strength theory). In fact, the contributions of $\Omega_{2}^{+}$ and $\Omega_{3}^{-}$ to offset each other, but the contributions of the interaction between $\Omega_{2}^{+}$ and $\Omega_{3}^{-}$ to cannot offset each other. Therefore, the fitted coefficients fully reflect both the physical and quantitative rationalities of how and how much *Ω*_2_ and *Ω*_3_ contribute to the equivalent stress *Ω_r_*. 

It should be noted that Equation (6) is in line with Yu’s unified strength theory and its final goal. In fact, Yu’s unified strength theory has touched this goal, since Equation (6) simply reflects a case in which *α* = 1 and *β* = 1/3 in Yu’s theory. Furthermore, Equation (6) has another theoretical basis: structural stressing state theory. Actually, Equation (6) is the result achieved by combining both Yu’s theory and structural stressing state theory, and is a typical result of interdisciplinary science.

### 5.3. Verification of Equation (6) with Natural Marble 

To verify the accuracy of Equation (6), Table 2 lists the equivalent stresses ($\Omega_{r}$) on the left side of Equation (6), and then compares their differences ($e_{\mathrm{fit}}$ is defined in Table 2) from the essential strength of concrete (Ω = 35 MPa). Equation (6) has an average error of only 0.02% (the average of the $e_{\mathrm{fit}}$ values). 

To verify whether Equation (6) is also valid for use with other brittle materials or not, this study conducted uniaxial and triaxial experiments with natural marble specimens and then obtained the essential strength and the combined principal stresses (Table 3). In Table 3, compressive stress is also positive; the essential strength of natural marble is Ω= 130.60 MPa; the size of the specimens is 50 mm × 50 mm × 100 mm; the sorting of the principal stresses adopts the rule in Equation (6). Evidently, even though the brittle marble specimens have considerable natural defects with respect to their material properties, the experiment also validates Equation (6), with an average error and a maximum error of −1.21% and 3.35%, respectively. This implies that Equation (6) has great applicability and omnipotence for brittle materials. 

### 5.4. Qualification of Equation (6) as the Formula for Strength

Now, the basic characteristics of Equation (6) can be summarized based on the verifications above. Equation (6) is obtained directly from experimental data, without the use of the preset assumptions on which the existing strength theories are based, and with only the use of suitable materials (homogeneous and isotropic materials). Therefore, Equation (6) could be an expression of the true physical properties of material strength and a reflection of the objective laws related to material strength. In other words, because the common stressing state mutation feature of specimens complies with the natural law stating that quantitative changes within a system will lead to qualitative changes within that system, both essential strength and Equation (6) naturally have a universality and generality that is suitable for brittle materials, as verified using brittle natural marble. Furthermore, the weight coefficients of Equation (6) embody the homogeneous and symmetrical contributions of principal stresses to the equivalent stress. In addition, the equivalent stress in Equation (6) only relates to the principal stress state, and does not include any material failure parameters related to the size effect. Meanwhile, equivalent stress and essential strength, with explicit physical meanings, are on the two sides of Equation (6), and their relation is described in a simple expression without any uncertain coefficients. Additionally, Equation (6) is generally conservative in quantity, since the essential strength is always equal to the equivalent stress, whose quantitative value is as high as the essential strength value. These characteristics of Equation (6) reflect the objectivity/truth, universality/generality, omnipotence, absoluteness/definiteness, homogeneity and symmetry, stability, simplicity and conservation of a physical law [49,50,51,52]. Therefore, Equation (6) can be qualified as the unified formula for the strength for brittle materials. As for whether or not Equation (6) is suitable for various materials (as a law of strength), further verification is required.

Accordingly, a general strength condition for brittle materials corresponding to Equation (6) can be set as

(8) $$\Omega_{1}-\frac{1}{4}\Omega_{2}-\frac{3}{4}\Omega_{3}\leq [\Omega ]$$

in which [Ω] is the allowable essential stress.

## 6. Conclusions

Through the experimental investigation into the uniaxial stressing state of concrete specimens, the essential strength of concrete, which is essentially without the size effect, was revealed based on the failure loads of the specimens with respect to their general stressing state mutation features, which are an embodiment of the natural law that quantitative change in a system will lead to qualitative change in that system. 

Then, biaxial and triaxial experiments of concrete specimens were carried out to obtain the data for combined principal stresses at the failure loads of specimens. Based on the data, the formula for strength was determined, revealing a definite relation between essential strength and combined principal stresses for concrete:

$$\Omega_{1}-\frac{1}{4}\Omega_{2}-\frac{3}{4}\Omega_{3}=\Omega$$

The experiments for the brittle natural marble validated the formula for strength. Correspondingly, a general strength condition can be proposed as:

$$\Omega_{1}-\frac{1}{4}\Omega_{2}-\frac{3}{4}\Omega_{3}\leq [\Omega ]$$

Furthermore, the definite relation between the essential uniaxial strength and the essential shear strength is achieved from the formula for strength:

$$\tau_{\Omega }=\frac{4}{7}\Omega$$

In conclusion, essential strength and the formula for strength could extend the traditional knowledge relating to material strength and could lead to the improvement of design codes on material strength. In addition, these discoveries still need deep and wide verifications of their rationality and validity, as well as investigation of their use for other materials, particularly their applications in practical engineering. In addition, the results achieved in this study cannot claim to have been able to resolve the size effect issue on material properties, but they explore a new way of dealing with this issue. 

## Figures and Tables

**Table 1:** Concrete specimens made in the uniaxial and compressive experiment.

Cube Specimens (mm) (Length × Width × Height)	Prism Specimens (mm) (Length × Width × Height)	Cylinder Specimens (mm) (Diameter × Height)
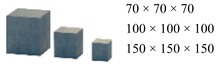	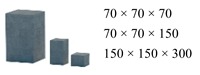	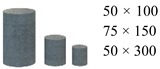

**Table 2:** Combined principal stresses and verification data (MPa).

$\Omega_{1}$	$\Omega_{2}$	$\Omega_{3}$	$\Omega_{r}$ ^a^	$e_{\mathrm{fit}}$ ^b^	$\Omega_{1}$	$\Omega_{2}$	$\Omega_{3}$	$\Omega_{r}$	$e_{\mathrm{fit}}$
37.53	10	0	35.03	0.09%	44.08	24	4	35.08	0.23%
38.05	14	0	34.55	−1.29%	45.05	28	4	35.05	0.14%
39.00	18	0	34.5	−1.43%	45.05	16	8	35.05	0.14%
40.35	22	0	34.85	−0.43%	46.01	20	8	35.01	0.03%
-given	26	0	35.47	1.34%	47.03	24	8	35.03	0.09%
41.95	30	0	34.45	−1.57%	52.04	20	16	35.04	0.11%
43.43	34	0	34.93	−0.20%	44.03	9	9	35.03	0.09%
40.03	8	4	35.03	0.09%	47.17	12	12	35.17	0.49%
41.41	12	4	35.41	1.17%	50.03	15	15	35.03	0.09%
42.04	16	4	35.04	0.11%	53.04	18	18	35.04	0.11%
43.03	20	4	35.03	0.09%	56.06	21	21	35.06	0.17%

Note: ^a^ $\Omega_{r}=\Omega_{1}-\Omega_{2}/4-3\Omega_{3}/4$ is the equivalent stress of Equation (8), which will be derived in the next section; ^b^ $e_{\mathrm{fit}}=[(\Omega_{r}-\Omega )/\Omega ]\times 100\%$ is the error between $\Omega_{r}$ and Ω = 35 MPa (the essential strength of concrete), which will be described below.

**Table 3:** Combined principal stresses of natural marble specimens and verifying data (MPa).

$\Omega_{1}$	$\Omega_{2}$	$\Omega_{3}$	$\Omega_{r}$	$e_{\mathrm{fit}}$	$\Omega_{1}$	$\Omega_{2}$	$\Omega_{3}$	$\Omega_{r}$	$e_{\mathrm{fit}}$
136.9	10	10	126.90	−2.83%	165.15	60	30	127.65	−2.26%
149.92	40	10	132.42	1.39%	179.72	100	30	132.22	1.24%
154.86	80	10	127.36	−2.48%	186.77	150	30	126.77	−2.93%
163.73	120	10	126.23	−3.35%	171.30	45	45	126.30	−3.29%
171.62	150	10	126.62	−3.05%	191.31	100	45	132.56	1.50%
178.83	180	10	126.33	−3.27%	202.36	150	45	131.11	0.39%
148.92	20	20	128.92	−1.29%	214.97	200	45	131.22	0.47%
160.14	70	20	127.64	−2.27%	202.00	68	68	134.00	2.60%
